# Elevated nitrogen allows the weak invasive plant *Galinsoga quadriradiata* to become more vigorous with respect to inter-specific competition

**DOI:** 10.1038/s41598-018-21546-z

**Published:** 2018-02-16

**Authors:** Gang Liu, Ying-Bo Yang, Zhi-Hong Zhu

**Affiliations:** College of Life Sciences, Shaanxi Normal University, 710119 Xi’an, P.R. China

## Abstract

Elevated nitrogen associated with global change is believed to promote the invasion of many vigorous exotic plants. However, it is unclear how a weak exotic plant will respond to elevated nitrogen in the future. In this study, the competitive outcome of a weak invasive plant (*Galinsoga quadriradiata*) and two non-invasive plants was detected. The plants were subjected to 3 types of culture (mixed, monoculture or one-plant), 2 levels of nitrogen (ambient or elevated at a rate of 2 g m^−2^ yr^−1^) and 2 levels of light (65% shade or full sunlight). The results showed that elevated nitrogen significantly promoted the growth of both the weak invader and the non-invasive plants in one-plant pots; however, growth promotion was not observed for the non-invasive species in the mixed culture pots. The presence of *G. quadriradiata* significantly inhibited the growth of the non-invasive plants, and a decreased negative species interaction was detected as a result of elevated nitrogen. Our results suggest that competitive interactions between *G. quadriradiata* and the non-invasive plants were altered by elevated nitrogen. It provides exceptional evidence that an initially weak invasive plant can become an aggressive invader through elevated nitrogen deposition.

## Introduction

Exotic species are commonly considered to be harmful to natural ecosystems of the introduced ranges^1,2^. Many aggressive invaders have received considerable attention from researchers worldwide, e.g., *Centaurea solstitialis*, *Mikania micrantha*, and *Bromus tectorum*^3–5^. Such species, which are jointly characterized by high competitive and colonizing abilities in natural communities^6,7^, usually have serious negative consequences, causing great concern. However, the effects of less aggressive exotic species have been consciously or unconsciously neglected by researchers for a long time. The invasive processes of such weak invasive species are poorly documented yet critical to understanding their expansion dynamics and effects in the future.

The high competitive ability of alien species has been suggested as a key factor associated with successful invasive potential, and competitive exclusion by native plant species seems to be a major force resisting exotic invasions^7^. It is commonly believed that the advantages of competitive ability account for the prevalence of vigorous invasive plants^6,7^. However, increasing evidence suggests that the competitive relationship between invasive and native plants varies with the invasion process^8,9^. For example, the dominance and negative effect of an initial aggressive invasive plant *Heracleum mantegazzianum* decreased with time^10^. Such a change is presumably attributed to adaptation and coevolution. Additionally, limited evidence suggests that the competitive relationship between invasive and native plants can also be changed by environmental variation, such as global change^11^. The magnitude of invasive species’ impacts on native plants may be modified by global changes that increase the availability of resources (e.g., nitrogen) and hence potentially alter the competitive dynamics between invasive and native species^12^. For instance, elevated nitrogen increased the predominance of invasive *Duchesnea indica* in contrast to native *Fragaria vesca*, hence environments with increased N deposition (i.e. from anthropogenic sources) could promote the invasive potential of *D. indica*^13^.

Nitrogen has been suggested as an important limiting factor to fast-growing plants, especially invasive species, including weak invaders (low competitive ability)^14^. In past decades, human activity has greatly increased the amount of biologically available N entering the natural environment in China^15^, and this trend is predicted to continue during the coming decades^16^. Such changes in nitrogen will certainly affect the competition between invasive and native plants. It has been suggested that vigorous invasive plants are usually more aggressive with respect to resource competition and are more effective at resource use compared to native plants^6,17^. Consequently, vigorous invasive plants will be promoted by elevated nitrogen deposition in the context of global change. Indeed, a growing body of evidence shows that elevated N deposition leads to a relative decline in native species biomass and a simultaneous increase in the biomass of vigorous invaders^18,19^. However, with our scope, no reports have dealt with how a weak invader and its competitors respond to elevated nitrogen.

*Galinsoga quadriradiata* is an annual herbaceous plant from Central and South America^20^. Although it has already colonized most abandoned land or farmland in Central, Eastern and Southern China^21,22^, and can decrease agricultural production by approximately 50% according to limited evidence^20^, *G. quadriradiata* is generally considered to be a relatively weak invader or weed due to its lack of high competitive and colonizing ability in natural communities. This species was first recorded in the “Flora of China” in 1979. However, its actual invasion history should be no less than 100 years according to local farmers’ descriptions. After such a long-term expansion, it has already reached almost all suitable climatic areas in China (unpublished material), and the habitat it invades is commonly restricted to farmland. Since China is one of ten mega-biodiversity countries in the world that play important roles in ensuring agriculture and food safety^23^, it is necessary to predict the future expansion of such an invader.

With the abovementioned shortfalls in mind, we conducted a greenhouse experiment to investigate the effect of elevated nitrogen on the competitive relationship between the weak invasive species *G. quadriradiata* and two confamilial competitors (*Heteropappus hispidus* and *Sonchus oleraceus*). Meanwhile, to mimic different light conditions in farmland and abandoned land, light source was also taken into consideration. We hypothesized that elevated nitrogen would allow the weak invader to become more vigorous and would change the relationship with its competitors. The present study addressed the two following questions: (1) How would the weak invasive plant and its competitors respond to elevated nitrogen? (2) Can elevated nitrogen or the synergistic effect of nitrogen and light change the competition outcome?

## Results

### Comparisons of harvested mass

**Figure 1:**
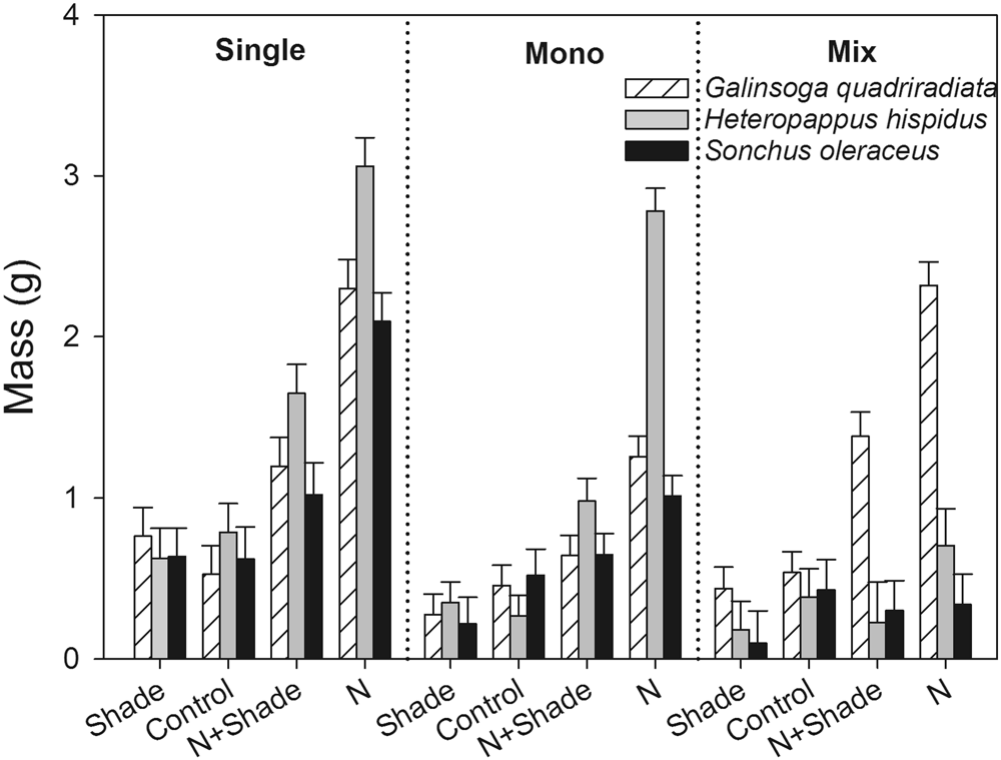
The harvested biomass (g) of the three species in each treatment. The horizontal axis represents nitrogen and light treatments: N+Shade, elevated nitrogen and 65% shaded; N, elevated nitrogen and natural sunlight; Shade, ambient nitrogen and 65% shaded; Control, ambient nitrogen and natural sunlight. Culture: Single, one-plant-culture; Mono, monoculture; Mix, Mixed culture. Values are means ± SE.

The result of the mixed model analysis revealed that Nitrogen (N), Light, Target species (Tsp) and Culture had significant effects on plant mass (Table 1). In general, the growth of the plants increased as a result of nitrogen addition but decreased with shade; the mass of the plants in the one-plant pots was generally higher than that in the monoculture and mixed culture. Moreover, interactive effects between or among some factors were also significant. For example, the interactions among Tsp, Culture, and N were significant. Compared with the mass of the individuals in the one-plant pots, the mass of the invader was not decreased by the mixed culture but was decreased in monoculture under elevated nitrogen (N+Shade and N) (Fig. 1). However, under elevated nitrogen, the mass of the native species *H. hispidus* in monoculture was not significantly different from that in the one-plant pots but was significantly higher than that in mixed culture; the mass of the exotic non-invasive species *S. oleraceus* in the one-plant pots was significantly higher than that in mono- and mixed-culture pots with elevated nitrogen. In pots without nitrogen addition (Shade and Control), the masses of both the invasive and non-invasive plants were generally not significantly affected by culture. In other words, the growth of the invader was not significantly inhibited by inter- or intra-specific competitors under the conditions without elevated nitrogen. The presence of invasive *G. quadriradiata* significantly inhibited the growth of the two non-invasive plants, especially when nitrogen was elevated. However, the presence of the two non-invasive species did not inhibit the growth of the invader regardless of whether nitrogen was added (Fig. 1).

**Table 1:** The dependence of total mass on experimental treatments based on mixed model ANOVA.

Effect	*df*	*F*	*P*
N	**1,165**	**242.9**	**<0.001**
Light	**1,165**	**74.6**	**<0.001**
N × Light	**1,165**	**46.0**	**<0.001**
Tsp	**2,165**	**15.9**	**<0.001**
Tsp × N	**2,165**	**11.9**	**<0.001**
Tsp × Light	2,165	2.5	0.090
Tsp × N × Light	**2,165**	**4.5**	**0.012**
Cul	**2,165**	**43.4**	**<0.001**
Cul × N	**2,165**	**10.8**	**<0.001**
Cul × Light	2,165	1.4	0.246
Cul × N × Light	**2,165**	**5.1**	**0.007**
Tsp × Cul	**4,165**	**18.8**	**<0.001**
Tsp × Cul × N	**4,165**	**13.1**	**<0.001**
Tsp × Cul × Light	4,165	1.3	0.291
Tsp × Cul × N × Light	**4,165**	**3.7**	**0.006**

Fixed factors: Nitrogen (N), Light, Target species (Tsp), and culture (Cul); Random factor: Pot number. Significant results are shown in bold.

Under the one-plant and monoculture scenarios, the total mass of the invader was not greater than those of the two non-invasive species in all treatments. However, in the mixed culture, the total mass of the invader was not lower than those of the two non-invasive species in all treatments (Fig. 1). Nitrogen elevation helped the invader outcompete the non-invasive competitors.

### Inter- and intra-specific competition intensity

**Figure 2:**
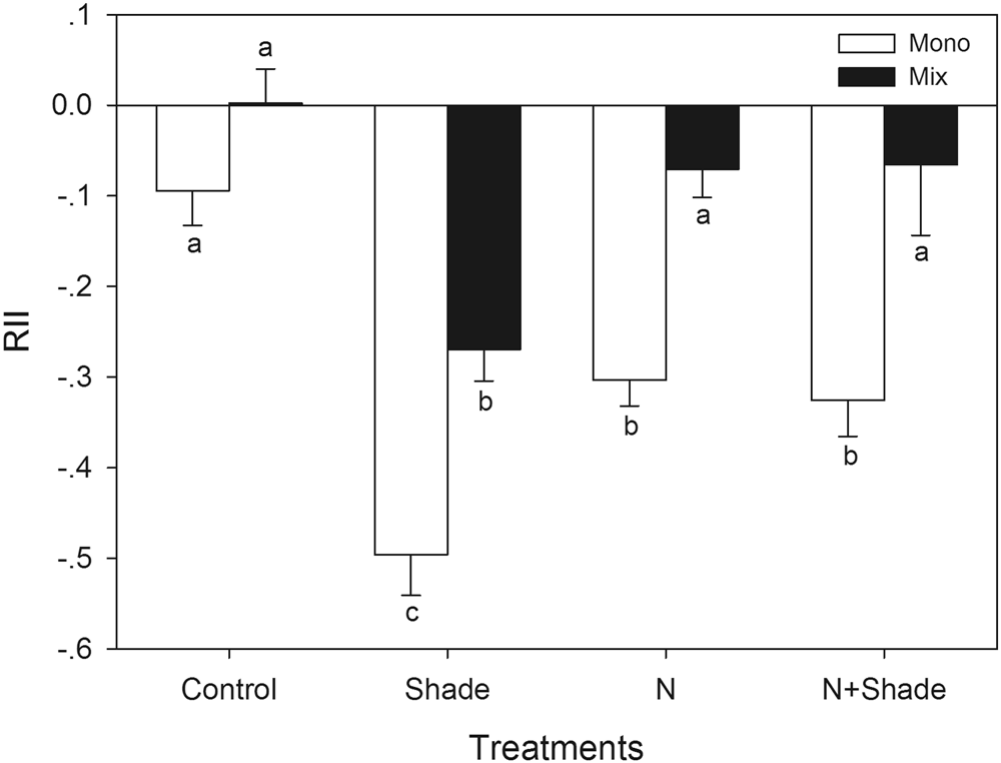
The inter- and intra-specific relative interaction index (RII) of *G. quadriradiata*. Culture type: Mix, Mixed culture; Mono, monoculture; Nitrogen and light treatments: N+Shade, elevated nitrogen and 65% shaded; N, elevated nitrogen and natural sunlight; Shade, ambient nitrogen and 65% shaded; Control, ambient nitrogen and natural sunlight. Values are means ± SE. Means with the same letter were not significantly different at an alpha level of 0.05.

The relative interaction index (RII) values were generally negative (Fig. 2). RII was significantly affected by Light (*F*_(1,188)_ = 25.86, *P* < 0.0001) and Culture (*F*_(1, 188)_ = 35.00, *P* < 0.0001). RII value was decreased under Shade treatment (Full sunlight, RII = −0.1187 ± 0.02392; Shade, RII = −0.2907 ± 0.02392). The intra-specific RII (monoculture) value of the invader was significantly lower than that of the inter-specific RII (mixed culture) (Fig. 2). There were significant interactive effects between Light and Nitrogen (*F*_(1,188)_ = 23.65, *P* < 0.0001). Shade treatment strengthened while elevated nitrogen alleviated inter- and intra-specific competition. Elevated nitrogen decreased the negative effect of the non-invasive plants on *G. quadriradiata* in mixed culture pots under light shaded conditions (mixed culture: Shade+N, RII = −0.07221 ± 0.04398; Shade, RII = −0.2696 ± 0.04248).

### Trait reaction norms under elevated nitrogen

**Figure 3:**
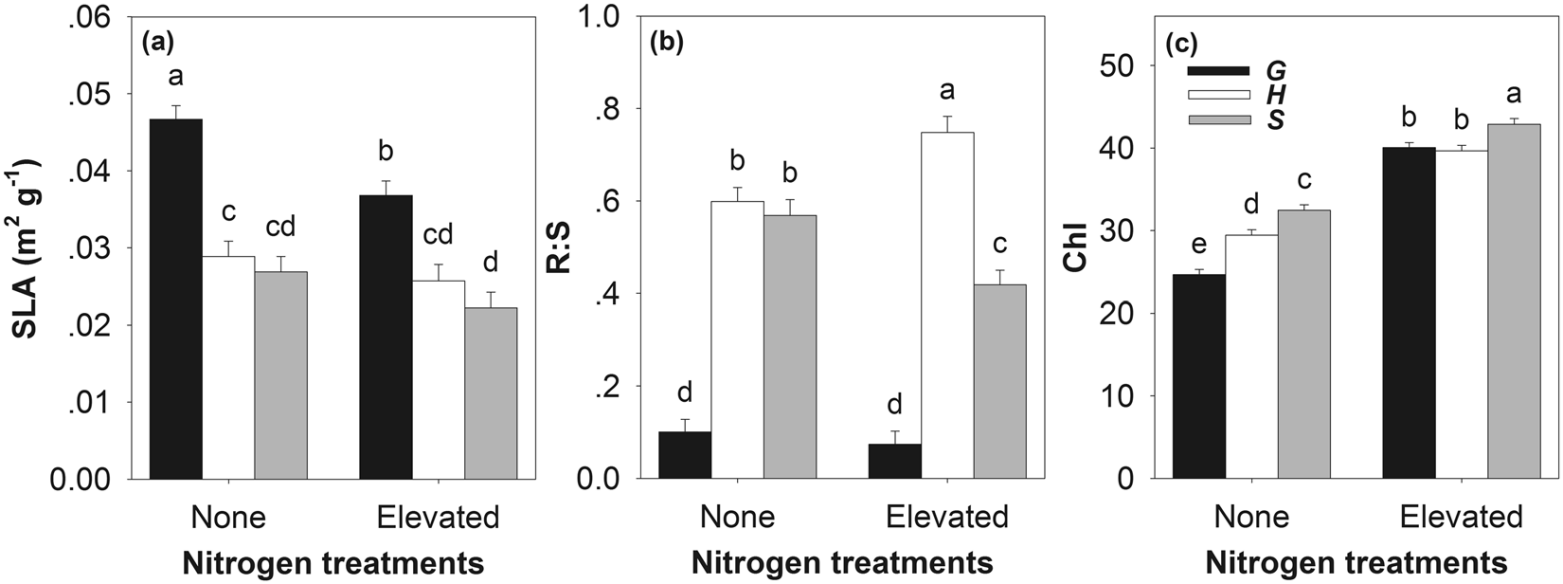
The specific leaf area (SLA, m^2^ g^−1^), root-shoot ratio (R:S) and leaf chlorophyll concentration (Chl, a unitless index from 0 to 100) of the three species under nitrogen treatments. Species: G, *Galinsoga quadriradiata*; H, *Heteropappus hispidus*; S, *Sonchus oleraceus*. Values are means ± SE. Means with the same letter were not significantly different at an alpha level of 0.05.

The specific leaf area (SLA) of the plant was significantly influenced by N, Light, Tsp and Culture, the root shoot ratio (R:S) of the plant was significantly influenced by Light and Tsp, and the leaf chlorophyll concentration (Chl) by N, Light, Tsp and Culture (Table 2, and see Supplementary Figs S1–S3). In general, *G. quadriradiata* exhibited lower R:S (*G. quadriradiata*, 0.087 ± 0.020; *H. hispidus*, 0.673 ± 0.023; *S. oleraceus*, 0.494 ± 0.023; *F*_(2,165)_ = 220.0, *P* < 0.001) and Chl (*G. quadriradiata*, 32.349 ± 0.437; *H. hispidus*, 34.53 ± 0.477; *S. oleraceus*, 37.663 ± 0.476; *F*_(2,84)_ = 36.1, *P* < 0.001) but higher SLA (*G. quadriradiata*, 0.042 ± 0.001; *H. hispidus*, 0.027 ± 0.002; *S. oleraceus*, 0.025 ± 0.001; *F*_(2,31)_ = 47.5, *P* < 0.001) compared with the two non-invasive species. There were significant interactive effects between N and Tsp on the R:S and Chl (except for SLA) (Table 2). Elevated nitrogen significantly decreased the SLA of the invader but did not significantly affect those of the native species (Fig. 3a). Elevated nitrogen did not change the R:S of the invader, but increased and decreased those of *H. hispidus* and *S. oleraceus*, respectively (Fig. 3b). Elevated nitrogen significantly increased Chl of all species. The Chl of the invader was significantly lower than those of the other two species in the pots without nitrogen addition. However, in pots with elevated nitrogen, the Chl of the invader was not lower than that of *H. hispidus* (Fig. 3c).

**Table 2:** The dependence of SLA, R:S, Chl, LCC, LNC, or C:N on experimental treatments based on mixed model ANOVA.

Effect	R:S			SLA			Chl			LCC			LNC		C:N	
	***df***	***F***	***P***	***df***	***F***	***P***	***df***	***F***	***P***	***df***	***F***	***P***	***F***	***P***	***F***	***P***
N	1,165	0.1	0.728	1,31	**13.3**	**0.001**	1,84	**460.3**	**<0.001**	1,30	**6.8**	**0.01**	**140.2**	**<0.001**	**114.7**	**<0.001**
Light	1,165	**110.1**	**<0.001**	1,31	**88.0**	**<0.001**	1,84	**12.0**	**0.001**	1,30	2.6	0.12	**175.2**	**<0.001**	**195.2**	**<0.001**
N × Light	1,165	2.0	0.157	1,31	0.5	0.494	1,84	**19.4**	**<0.001**	1,30	0.0	0.95	**37.1**	**<0.001**	0.9	0.357
Tsp	2,165	**220.0**	**<0.001**	2,31	**47.5**	**<0.001**	2,84	**36.1**	**<0.001**	2,30	**34.4**	**<0.001**	**44.2**	**<0.001**	**78.4**	**<0.001**
Tsp × N	2,165	**10.8**	**<0.001**	2,31	1.7	0.201	2,84	**11.5**	**<0.001**	2,30	0.0	0.99	**5.9**	**0.007**	**21.3**	**<0.001**
Tsp × Light	2,165	**24.5**	**<0.001**	2,31	2.8	0.073	2,84	1.9	0.159	2,30	0.7	0.49	**8.8**	**0.001**	**18.6**	**<0.001**
Tsp × N × Light	2,165	**11.9**	**<0.001**	2,31	1.6	0.226	2,84	**15.9**	**<0.001**	2,30	0.1	0.93	**7.0**	**0.003**	**7.2**	**0.003**
Cul	2,165	**11.8**	**<0.001**	2,31	**13.0**	**<0.001**	2,84	**20.9**	**<0.001**	2,30	1.2	0.33	**3.4**	**0.048**	1.5	0.238
Cul × N	2,165	**4.6**	**0.011**	2,31	1.0	0.376	2,84	0.9	0.395	2,30	0.5	0.63	**6.3**	**0.005**	**13.6**	**<0.001**
Cul × Light	2,165	**4.1**	**0.019**	2,31	**13.4**	**<0.001**	2,84	0.4	0.663	2,30	0.3	0.73	**6.0**	**0.006**	**7.1**	**0.003**
Cul × N × Light	2,165	**9.6**	**<0.001**	2,31	1.4	0.267	2,84	1.8	0.175	2,30	0.7	0.49	**3.7**	**0.038**	**3.7**	**0.036**
Tsp × Cul	4,165	**8.7**	**<0.001**	4,31	2.4	0.072	4,84	**3.4**	**0.013**	4,30	1.0	0.42	**4.1**	**0.009**	**4.0**	**0.011**
Tsp × Cul × N	4,165	**6.7**	**<0.001**	4,31	0.6	0.655	4,84	2.1	0.087	4,30	0.8	0.53	**5.1**	**0.003**	**10.7**	**<0.001**
Tsp × Cul × Light	4,165	**9.6**	**<0.001**	4,31	0.8	0.541	4,84	**3.0**	**0.023**	4,30	0.2	0.96	**3.2**	**0.027**	1.4	0.261
Tsp × Cul × N × Light	4,165	**9.5**	**<0.001**	4,31	0.6	0.665	4,84	2.1	0.086	4,30	1.4	0.27	**2.8**	**0.042**	**4.1**	**0.010**

SLA, specific leaf area (m^2^ g^−1^); R:S, root-shoot ratio; Chl, leaf chlorophyll concentration (a unitless index from 0 to 100); LCC, leaf carbon concentration (%); LNC, leaf nitrogen concentration (%); C:N, leaf carbon-nitrogen ratio. Fixed factors: Nitrogen (N), Light, Target species (Tsp), and culture (Cul); Random factor: Pot number. Significant results are shown in bold.

**Figure 4:**
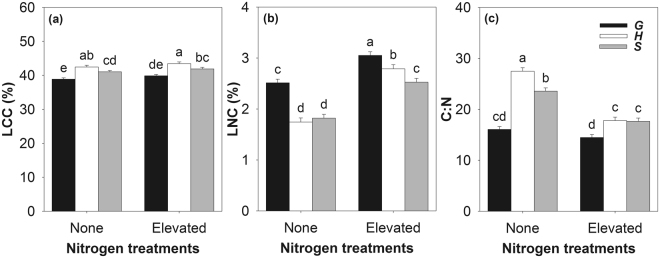
The leaf carbon concentration (LCC, %), leaf nitrogen concentration (LNC, %) and leaf carbon-nitrogen ratio (C:N) of the three species under nitrogen treatments. Species: G, *Galinsoga quadriradiata*; H, *Heteropappus hispidus*; S, *Sonchus oleraceus*. Values are means ± SE. Means with the same letter were not significantly different at an alpha level of 0.05.

The leaf carbon concentration (LCC) of the plant was significantly influenced by N and Tsp, the leaf nitrogen concentration (LNC) by N, Light, Tsp and Culture, and the leaf carbon-nitrogen ratio (C:N) by N, Light and Tsp (Table 2, and see Supplementary Figs S4–S6). In general, nitrogen addition increased the LCC and LNC but decreased the C:N of the plants. There were significant interactive effects between N and Tsp on LNC and C:N (except for LCC). The LNCs of both the invader and the native species were significantly increased by nitrogen treatment (Fig. 4b). The C:N of the invasive plant was not significantly changed by nitrogen treatment, while those of the non-invasive plants were decreased by nitrogen treatment (Fig. 4c). The invasive plant had higher LNC but lower C:N than the other two species (Fig. 4b and c).

## Discussion

Our study found that elevated nitrogen altered the competitive relationship between the weak invader *G. quadriradiata* and the two non-invasive plant species. It indicates that elevated nitrogen deposition may strongly influence the invasion processes of weak invaders as well as vigorous invasive plants in natural ecosystem. We should be especially cautious of the expansion of weak invasive plants in response to elevated nitrogen deposition in the context of global change. The results showed that the competitive interactions between *G. quadriradiata* and the non-invasive plant species were not altered by light treatment but by nitrogen enrichment. In the Control and Shade treatments, when we planted the weak invader together with the two non-invasive species, no competitive advantage was exhibited. However, in the treatments with elevated nitrogen (N and N+Shade), the weak invader obtained a considerable advantage and outcompeted the non-invasive plants (Fig. 1). The shade treatment suppressed the growth of both the invader and non-invasive species, especially in pots with elevated nitrogen (Fig. 1). However, it did not change the competitive pattern between *G. quadriradiata* and the non-invasive species. The results indicate that initially weak invasive plants can become more vigorous under elevated nitrogen deposition associated with global change. As suggested by numerous studies, the invasion of vigorous invasive plants can usually be accelerated by elevated nitrogen^3,19,24^. Such vigorous invasive plants are commonly characterized by a conspicuous and strong competitive ability with respect to resources^6^. However, *G. quadriradiata* is not a vigorous, but rather weak, competitor under ambient conditions (Fig. 1). Although *G. quadriradiata* is a worldwide invasive plant in farmland, it is generally considered to be a relatively weak invader or weed due to its lack of high competitive and colonizing ability in natural communities which is usually characterized by relatively low soil nitrogen resource and high competition from close relatives. Thus, our results suggest that competition relationships between some originally weak exotic species and their competitors can be changed by elevated nitrogen. Such changes of competition outcome caused by nitrogen resource were also occasionally reported by some researchers^13^. This suggests that necessary attention should be paid to the expansion of invasive plants which are weak under low nitrogen but powerful under rich nitrogen conditions in the context of global change.

In fact, elevated nitrogen significantly promoted the growth of both the weak invader and the non-invasive plants in the one-plant pots; however, such a promotional effect was not observed for the non-invasive species in the mixed culture pots (Fig. 1 and Table 1). Moreover, a decreased negative species interaction was detected as a result of elevated nitrogen, especially in Shade treatment (Fig. 2). The interactions between the invader and the two competitors were generally negative, and the intra-specific RIIs of the invader were significantly lower than the inter-specific RIIs in most of the treatments (Fig. 2). Taken together, the results suggest that, the negative competitive effect of *G. quadriradiata* counteracts the positive effect of nitrogen elevation on the growth of the non-invasive plants, and that elevated nitrogen alleviated the negative effects of the non-invasive plants on *G. quadriradiata*. However, the high intra-specific RII value suggested intense intra-specific competition in the invader’s population. Previous studies suggested that increased nitrogen deposition alleviated, enhanced or even had no effect on the competitive effects of invasive plants on their competitors^3,25,26^. Our results revealed that elevated nitrogen enhanced the competitive effect of *G. quadriradiata* on its competitors. Many cases have underlined thel roles of competition for nutrient resources during plants’ invasion process^27^. It is indeed crucial to examine resource competition dynamics and the impact of global environmental change on competitive interactions between invasive and native species^28^. The conflicting results suggest that the response of invaders and native competitors may be species-specific.

Changes in the availability of resources associated with plant invasions may thus create conditions that may either increase or decrease the competitive ability of invasive species in contrast to those of native or other alien species^28,29^. How these changes will affect resource competition between alien and native species is key to improving our understanding of the long-term effects of plant invasions on native communities^28^. Global change is believed to alter the competitive relationship between invasive and native species in many ways, such as through land cover change, elevated CO_2_ and the nitrogen fertilization effect^11,29^. All these factors can enhance environmental resource availability, and nitrogen is especially important for invasive plants. This is probably because nitrogen is crucial for fast-growing plants and is usually difficult to obtain under natural conditions (except for nitrogen-fixing species)^30,31^. However, as a result of global change, nitrogen may be captured more easily by plants than before. In fact, elevated nitrogen can occur as a result of atmospheric nitrogen deposition as well as anthropogenic activities, such as agricultural fertilization of farmland^13^. The nitrogen resource of wild field can directly or indirectly be enhanced by such agricultural activities. In China, the large area of farmland and frequent agricultural fertilization^32^ may unintentionally further accelerate the colonization and expansion of *G. quadriradiata* into natural plant communities.

Morphological traits are suggested to be directly associated with plant competitive ability^33^. The R:S of the invader was significantly lower than that of the non-invasive competitor, and it was not changed by nitrogen, light or culture type (Fig. 3b and Table 2). This indicates that the weak competitive ability of *G. quadriradiata* in the Control treatment may be attributed to its lower root allocation, which has been suggested as a main cause for failure in soil nutrient competition^34^. However, under the scenario of elevated nitrogen, a low R:S may help plants to increase biomass accumulation of aboveground, hence resulting in a competitive advantage^13^. Other species with a low R:S have also been reported to successfully invade into nutrient-rich habitats^13,35^. Although nitrogen addition decreased the SLA of all species, that of *G. quadriradiata* was still higher than those of the native competitors (Fig. 4a). Elevated nitrogen increased the Chl of the invader as well as those of the non-invasive plants (Fig. 4c). It should be noted that the invader obtained highest rate of increase compared to the two native species (*G. quadriradiata*, *H. hispidus* and *S. oleraceus* increased by 53.96%, 30.76% and 28.76% respectively). Higher SLA species invest less dry matter per leaf, and have shorter leaf life spans^36^. These are associated with lower leaf construction cost, higher nitrogen allocation to photosynthesis, and higher photosynthetic nitrogen use efficiency^37,38^. The advantages associated with SLA and photosynthetic traits (e.g., chlorophyll concentration) subsequently account for the vigorous invasiveness of exotics^37,39,40^. In the present case, *G. quadriradiata* maintained its R:S at lower level and SLA at higher level, and increased chlorophyll concentration in response to elevated nitrogen. Moreover, the invasive plant had higher LNC but lower C:N than the other two species (Fig. 4). Higher leaf nitrogen content is usually related to higher assimilation rate, and is subsequently associated with higher growth rate of plant shoot and higher competitive ability on aboveground resource^13^. The C:N balance, is also important for regulation of plant growth and development^41^. For example, C:N plays an important role in regulating leaf senescence^42^. Plants with lower C:N ratio are usually characterized by higher growth rate^43^, and subsequently successful invasive plants are commonly characterized by lower C:N compared to non-invasive plants^17^. In sum, these attributes may help *G. quadriradiata* successfully outcompete the two competitors. However, it should be acknowledged that the present one-year experiment can not definitely uncover the invasion process of *G. quadriradiata* in the future. Further studies involving multiple generation under field conditions are necessary to reach a solid conclusion about this issue.

## Methods

### Experimental design

Three species of the Asteraceae family, i.e., the invasive species *G. quadriradiata* and the native species *H. hispidus* and *S. oleraceus* (which is native to Eurasia, although some researchers take it as a naturalized species in China), were chosen for comparison. All seeds we used in this study were collected from the Qinling-Bashan Mountains in 2014, and were stored in a 4 °C refrigerator.

In early May 2015, the seeds of the three species were sown in nursery pots. Two weeks later, when the seedlings were approximately 3 cm in height, they were transplanted into plastic pots (diameter 12 cm; height 10 cm). The pots were filled with sand and soil in a 1:1 proportion by volume. Two types of culture (Mixed and Monoculture) were conducted. For the mixed-culture, one invasive plant and one of the two non-invasive species were planted in the same pot. For the monoculture, two *G. quadriradiata*, two *H. hispidus*, or two *S. oleraceus* seedlings were planted together. Additionally, to compare the inter- and intra-specific competition intensity, we established a one-plant-culture treatment for each species, i.e., one seedling of each species was transplanted into one pot. After transplanting, all pots were subjected to two environmental treatments based on a factorial design: nitrogen (ambient or elevated) and light (shade or full sunlight) with 10 replicates. For the nitrogen treatments, each pot was watered with 5 ml deionized water (ambient) or 3.783 g L^−1^ NH_4_NO_3_ solution (elevated). The nitrogen addition simulated the current nitrogen deposition rate of 2 g m^−2^ yr^−1^ in China^15^. For the Shade treatment, the radiation intensity was controlled at 65% of the natural level. There were 320 pots and 520 individuals in total. All pots were randomly arranged in an 80 m^2^ greenhouse to avoid the influence of herbivores, and were watereddaily to keep the soil wet.

Two months later, when the invasive plants were almost mature, the leaf chlorophyll concentration (Chl) of the invasive species was measured using a portable chlorophyll meter SPAD-502 (Spectrum Technologies, Inc., Plainfield, IL, U.S.). The mean of three readings from the chlorophyll meter was calculated from 3 fully expanded leaves. In mid-September 2015, all parts of the plants were harvested and leaf areas were measured immediately. The dry weight of the root, leaf, and stem of each individual was separately measured after the samples had been dried to a constant weight in an oven at 60 °C. Elemental analysis for leaf carbon concentration (LCC, %) and leaf nitrogen concentration (LNC, %) was performed on Vario EL cube CHNOS elemental analyzer (Elementar Analysen Systeme, Hanau, Germany). Five individuals’ leaf sample of each species under each treatment were randomly chosen, and thus a total of 200 sample were measured.

### Relative interaction index (RII)

The relative interaction index (RII) between *G. quadriradiata* and the native or exotic competitors was calculated according to the following equation^44^:

1 $${\rm{RII}}=({{\rm{M}}}_{1}-{{\rm{M}}}_{0})/{({\rm{M}}}_{1}+{{\rm{M}}}_{0})$$

M_1_ is the mass of *G. quadriradiata* under mixed culture or monoculture conditions. M_0_ is the mass of *G. quadriradiata* in the one-plant pots. When calculated using data from the mixed culture treatments, the RII represents the intensity of inter-specific competition between the invasive and non-invasive species. When calculated using data from the monoculture treatments, the RII represents the intensity of intra-specific competition of *G. quadriradiata*. RII ranges from −1 to 1. if RII < 0, the plant experiences a negative impact from its competitor; if RII > 0, the plant experiences a positive impact from its competitor; if RII = 0, no impact is observed^44^.

### Data analyses

R:S was calculated as the ratio between root dry mass and aboveground dry mass. Specific leaf area (SLA) is defined as the ratio of leaf area to dry mass. C:N was calculated as the ratio between LCC and LNC. A mixed model was used to evaluate the effects of the independent variables of Nitrogen (ambient or elevated N), Light (Shade or Full sunlight) Culture (one-plant, mixed or monoculture), and Target species (*G. quadriradiata*, *H. hispidus* or *S. oleraceus*) on the dependent variables mass, R:S, SLA, Chl, LCC, LNC and C:N respectively. Pot number was used as the random factor of the model. All analyses were conducted using SAS 9.3 (SAS Institute Inc., Cary, NC, USA).

### Data availability statement

All data generated or analyzed during this study are included in this published article.

## Electronic supplementary material

Supplementary Figures
