# Supplementary material for: Elevated nitrogen allows the weak invasive plant Galinsoga quadriradiata to become more vigorous with respect to inter-specific competition
Source: Sci Rep. 2018 Feb 16;8:3136. doi: 10.1038/s41598-018-21546-z (PMC5816611; doi:10.1038/s41598-018-21546-z)
Supplement: Supplementary file 1 — Supplementary Figures [file 41598_2018_21546_MOESM1_ESM.docx]

# Elevated nitrogen allows the weak invasive plant *Galinsoga quadriradiata* to become more vigorous with respect to inter-specific competition

Gang Liu, Yingbo Yang, Zhihong Zhu*

College of Life Sciences, Shaanxi Normal University, 710119 Xi'an, P. R. China

*Corresponding author

E-mail: zhuzhihong@snnu.edu.cn

**Supplementary Figures:**

**
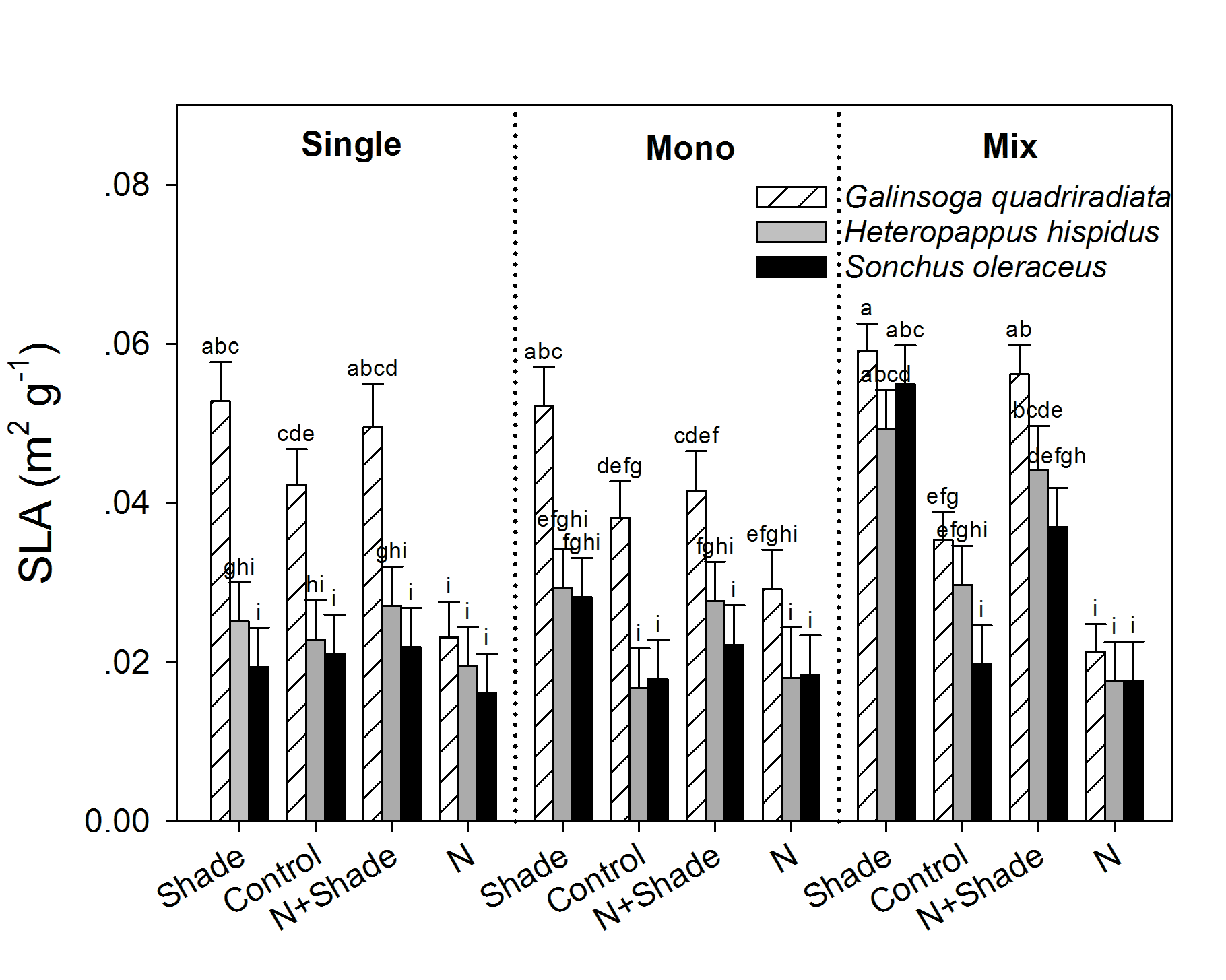
**

**Supplementary Figure S1. The specific leaf area (SLA, m^2^ g^-1^) of the three species in each treatment.** The horizontal axis represents nitrogen and light treatments: N+Shade, elevated nitrogen and 65% shaded; N, elevated nitrogen and natural sunlight; Shade, ambient nitrogen and 65% shaded; Control, ambient nitrogen and natural sunlight. Culture: Single, one-plant-culture; Mono, monoculture; Mix, Mixed culture. Values are means ± SE. Means with the same letter were not significantly different at an alpha level of 0.05.

**
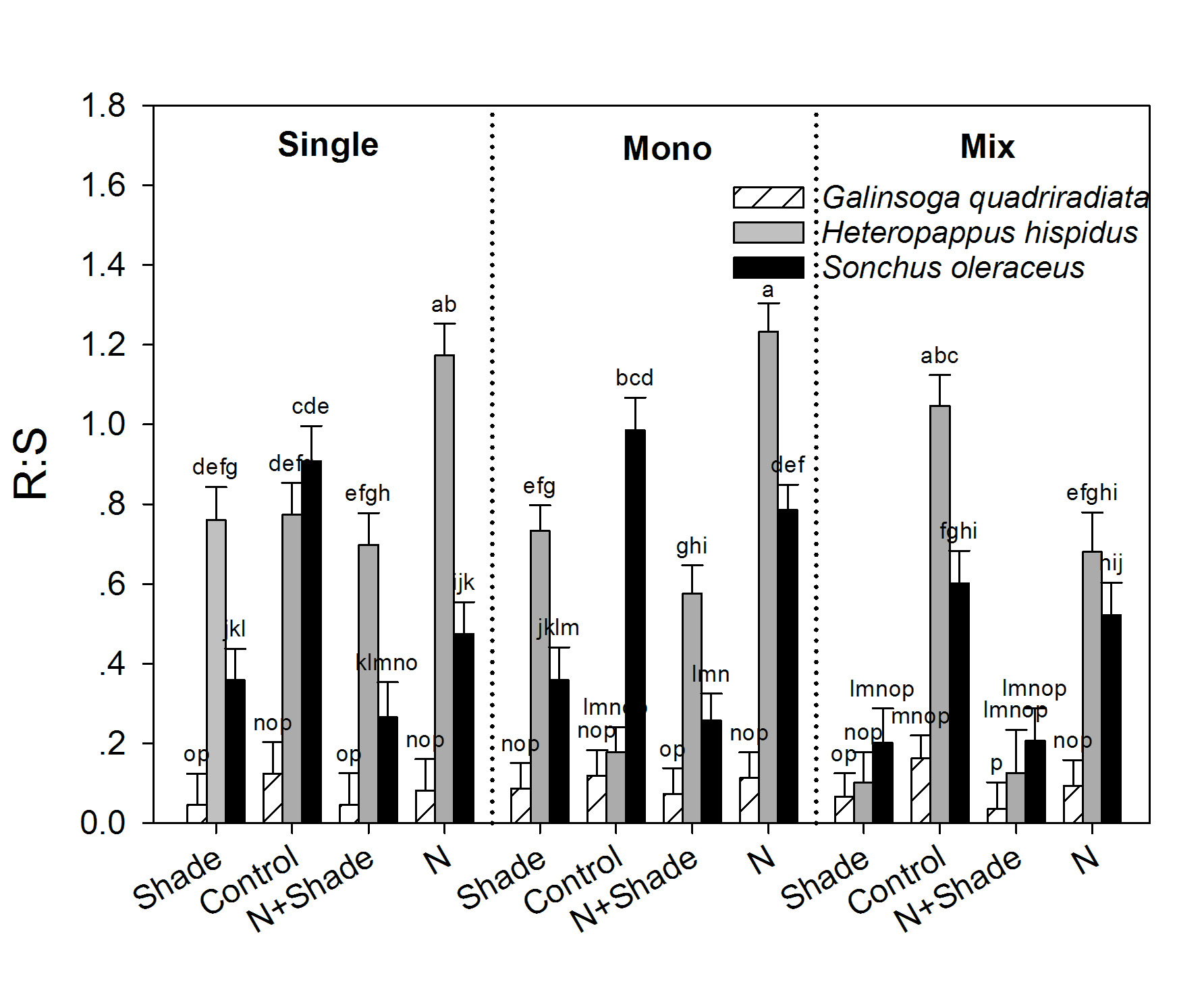
**

**Supplementary Figure S2. The root-shoot ratio (R:S) of the three species in each treatment.** The horizontal axis represents nitrogen and light treatments: N+Shade, elevated nitrogen and 65% shaded; N, elevated nitrogen and natural sunlight; Shade, ambient nitrogen and 65% shaded; Control, ambient nitrogen and natural sunlight. Culture: Single, one-plant-culture; Mono, monoculture; Mix, Mixed culture. Values are means ± SE. Means with the same letter were not significantly different at an alpha level of 0.05.

**
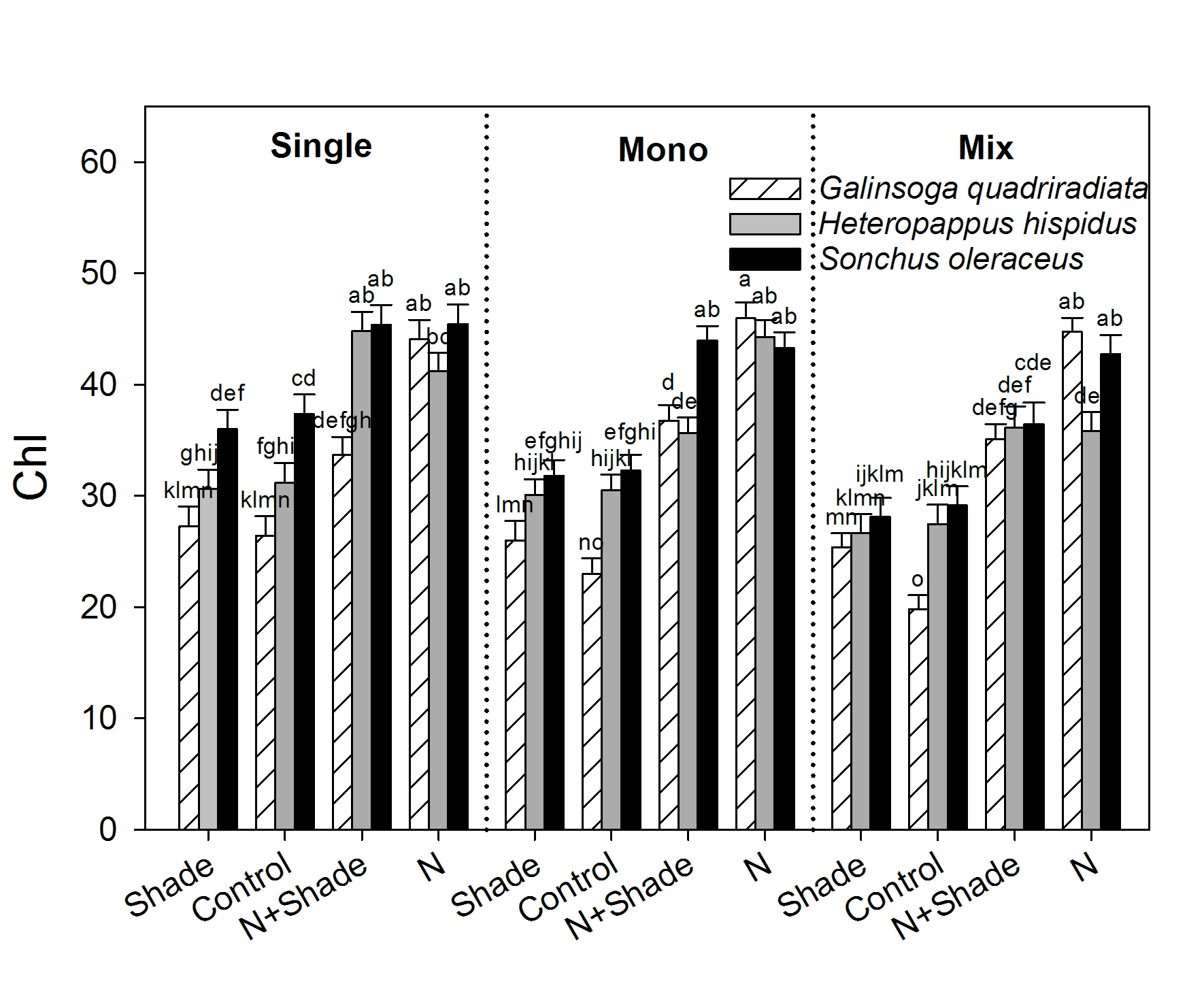
**

**Supplementary Figure S3. The leaf chlorophyll concentration (Chl, a unitless index from 0 to 100) of the three species in each treatment.** The horizontal axis represents nitrogen and light treatments: N+Shade, elevated nitrogen and 65% shaded; N, elevated nitrogen and natural sunlight; Shade, ambient nitrogen and 65% shaded; Control, ambient nitrogen and natural sunlight. Culture: Single, one-plant-culture; Mono, monoculture; Mix, Mixed culture. Values are means ± SE. Means with the same letter were not significantly different at an alpha level of 0.05.


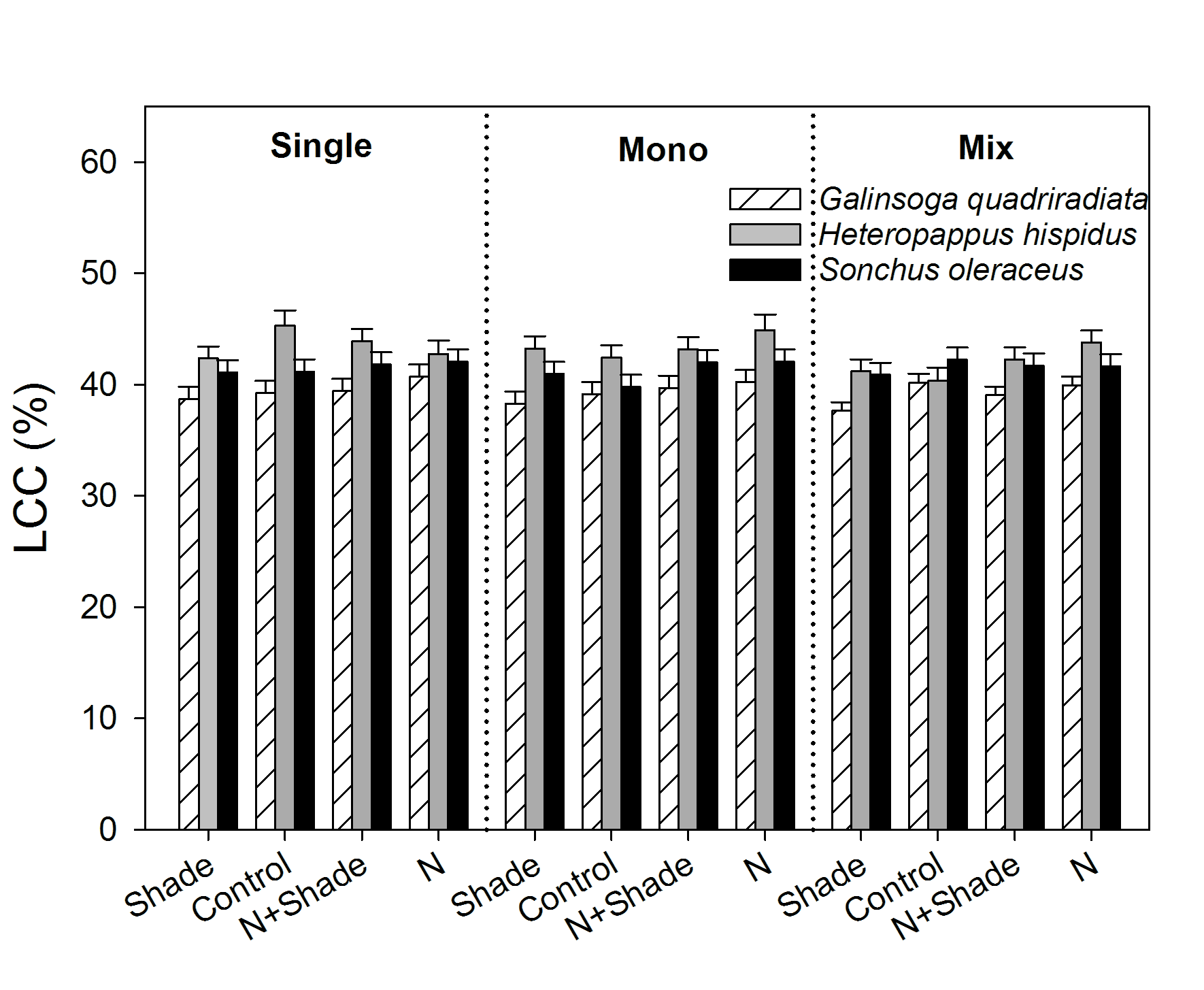


**Supplementary Figure S4. The leaf carbon concentration (LCC, %) of the three species in each treatment.** The horizontal axis represents nitrogen and light treatments: N+Shade, elevated nitrogen and 65% shaded; N, elevated nitrogen and natural sunlight; Shade, ambient nitrogen and 65% shaded; Control, ambient nitrogen and natural sunlight. Culture: Single, one-plant-culture; Mono, monoculture; Mix, Mixed culture. Values are means ± SE.


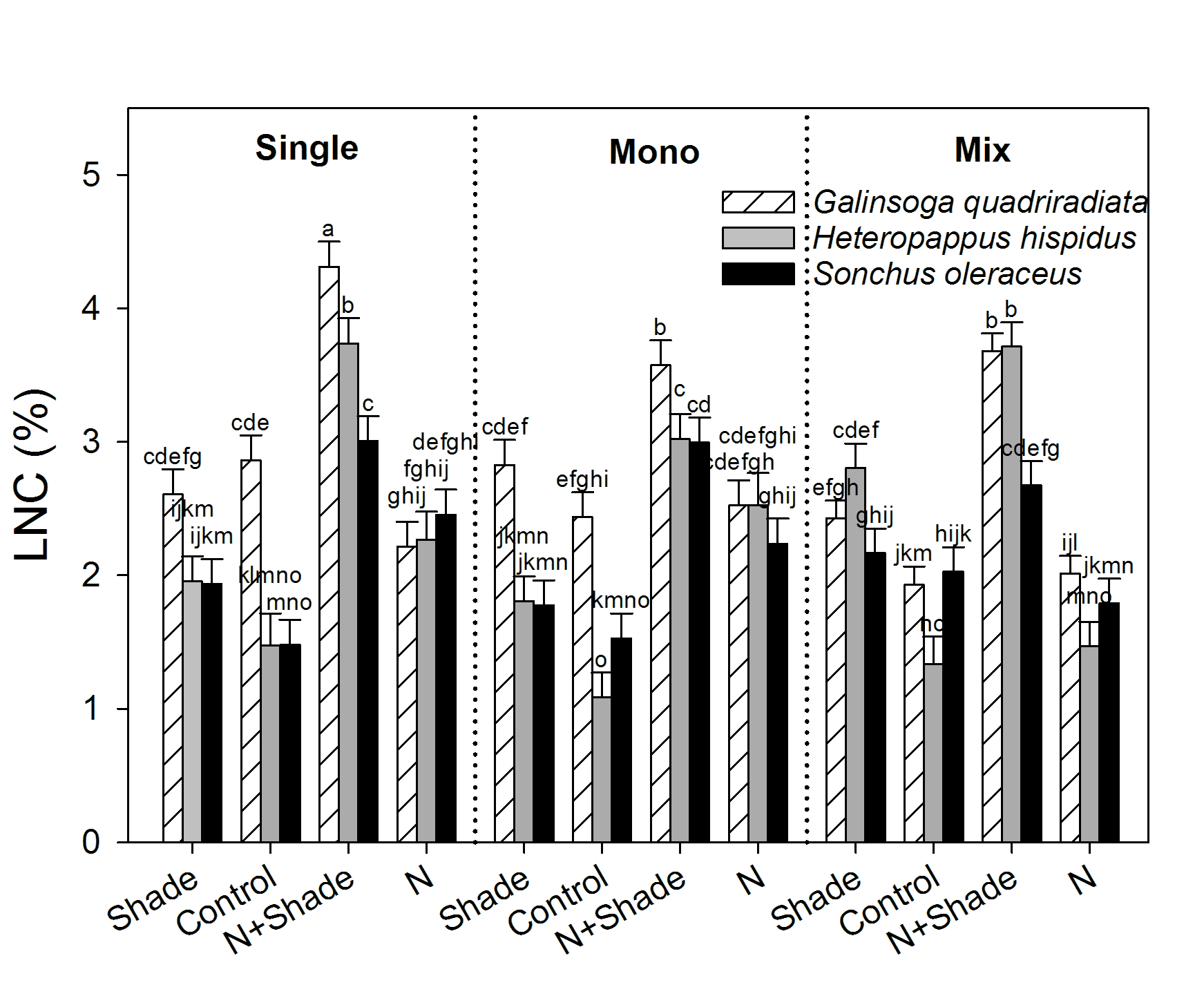


**Supplementary Figure S5. The leaf nitrogen concentration (LNC, %) of the three species in each treatment.** The horizontal axis represents nitrogen and light treatments: N+Shade, elevated nitrogen and 65% shaded; N, elevated nitrogen and natural sunlight; Shade, ambient nitrogen and 65% shaded; Control, ambient nitrogen and natural sunlight. Culture: Single, one-plant-culture; Mono, monoculture; Mix, Mixed culture. Values are means ± SE. Means with the same letter were not significantly different at an alpha level of 0.05.


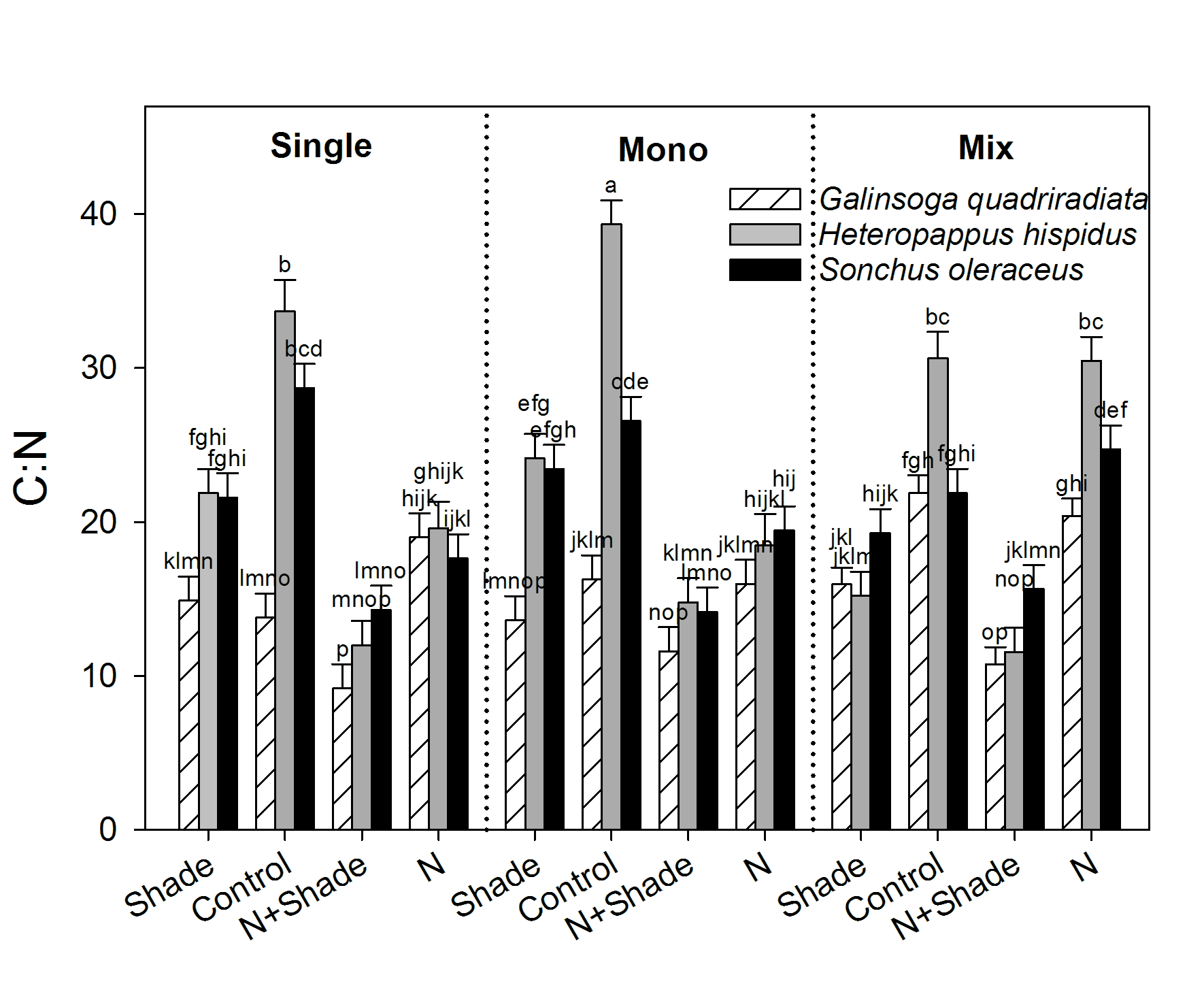


**Supplementary Figure S6. The leaf carbon to nitrogen ratio (C:N) of the three species in each treatment.** The horizontal axis represents nitrogen and light treatments: N+Shade, elevated nitrogen and 65% shaded; N, elevated nitrogen and natural sunlight; Shade, ambient nitrogen and 65% shaded; Control, ambient nitrogen and natural sunlight. Culture: Single, one-plant-culture; Mono, monoculture; Mix, Mixed culture. Values are means ± SE. Means with the same letter were not significantly different at an alpha level of 0.05.
